# Assessing Measles Transmission in the United States Following a Large Outbreak in California

**DOI:** 10.1371/currents.outbreaks.b497624d7043b1aecfbfd3dfda3e344a

**Published:** 2015-05-07

**Authors:** Seth Blumberg, Lee Worden, Wayne Enanoria, Sarah Ackley, Michael Deiner, Fengchen Liu, Daozhou Gao, Thomas Lietman, Travis Porco

**Affiliations:** St. Mary's Medical Center, San Francisco, California, USA; FI Proctor Foundation, UCSF, San Francisco, California, USA; Fogarty International Center, NIH, Bethesda, Maryland, USA; FI Proctor Foundation, UCSF, San Francisco, California, USA; FI Proctor Foundation, UCSF, San Francisco, California, USA; Department of Epidemiology & Biostatistics, UCSF, San Francisco, California, USA; FI Proctor Foundation, UCSF, San Francisco, California, USA; Department of Epidemiology & Biostatistics, UCSF, San Francisco, California, USA; FI Proctor Foundation, UCSF, San Francisco, California, USA; FI Proctor Foundation, UCSF, San Francisco, California, USA; FI Proctor Foundation, UCSF, San Francisco, California, USA; Department of Ophthalmology, UCSF, San Francisco, California, USA; Department of Epidemiology & Biostatistics, UCSF, San Francisco, California, USA; FI Proctor Foundation, UCSF, San Francisco, California, USA; Department of Ophthalmology, UCSF, San Francisco, California, USA; Department of Epidemiology & Biostatistics, UCSF, San Francisco, California, USA

**Keywords:** effective reproduction number, infectious diseases, Measles, transmission chain

## Abstract

The recent increase in measles cases in California may raise questions regarding the continuing success of measles control. To determine whether the dynamics of measles is qualitatively different in comparison to previous years, we assess whether the 2014-2015 measles outbreak associated with an Anaheim theme park is consistent with subcriticality by calculating maximum-likelihood estimates for the effective reproduction numbe given this year’s outbreak, using the Galton-Watson branching process model. We find that the dynamics after the initial transmission event are consistent with prior transmission, but does not exclude the possibilty that the effective reproduction number has increased.

## Introduction

A recent outbreak of measles linked with one or more Disney theme parks has reinvigorated discussion of measles vaccination.^1^^,^ 2 ^,^ 18 By April 10, 2015, 131 confirmed measles cases were reported in the outbreak in California, with 40 cases in California having traveled to the parks in December.^3^^,^ 16 An additional 16 cases linked to this outbreak have been reported elsewhere in the US, in six other states^12^ by April 10, 2015, for a total of 147.^1^ In Quebec, a total of 158 cases of the same genotype (B3) were reported in a non-immunizing religious community^13^ by March 28, and the importation to Canada is linked to travel to Disneyland. Cases from this outbreak also were observed in Mexico.^12^ This outbreak, larger than those typically seen in recent years in North America, may raise questions regarding the continuing success of measles control.

Between 2001 and 2011, the average number of individuals who became infected by one infectious case (the effective reproduction number, *R_eff_*) in the United States was estimated to be 0.52 individuals (95% CI: 0.44, 0.60).^4^ If *R_eff_* is less than one, transmission is subcritical; each infectious case, on average, directly infects fewer than one person; all outbreaks die out under subcritical conditions.^19^ Thus, our prior findings implied that measles could not sustain itself in the United States. Here, we use the 131 confirmed cases from the California outbreak to assess whether *R_eff_* has increased significantly.

## Methods

We used the Galton-Watson branching process model with our previously inferred dispersion parameter for measles, supplemented by sensitivity analysis. The number of new cases caused by each case is modeled by the negative binomial distribution, with parameters *R*_eff_ and a dispersion parameter *k.* 6 ^,^ 17 The dispersion parameter permits explicit modeling of heterogeneity of disease transmission as occurs when superspreading is present. Estimates of these two parameters for the US, as well as the rate of introduction of measles into the US, were used to assess the current outbreak.

While heterogeneity and superspreading are key features of measles which must be taken into account, analysis of transmission at the very beginning of the epidemic—in the theme park—is subject to severe selection bias^5^; analysis of an outbreak chosen specifically because of its size can hardly yield other than an upwardly biased assessment of transmission. Moreover, the relatively quiet years from 2001 and 2011 (where cluster data are available) may not be sufficient to fully characterize the tail of the transmission distribution. Instead of modeling the initial transmission in the theme park, we instead follow the subsequent transmission from cases exposed in Disneyland. These subsequent cases may be considered a kind of natural experiment in seeding measles throughout the state, while recognizing that contacts of cases exposed at the theme park may systematically differ from the California population. Because the number of California cases exposed in Disneyland is known to be 40, we used California case counts to derive a current estimate of the effective reproduction number.

Our analysis of the epidemic using the Galton-Watson process will assume a negative binomial distribution for the number of secondary cases caused by each active case. Assuming the negative binomial distribution 6 ^,^ 17 for the number of new cases caused by each case in one generation, the probability of *i* cases causing *j* cases in the subsequent generation is 7


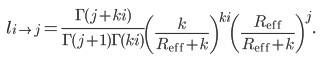


These *j* cases go on to cause, in general, further cases. If eventually transmission terminates, with no new cases arising in subsequent generations, we can compute the total number of cases so far, i.e., the total cluster size. The probability of *i* cases causing a cluster of size *j* is given by 7

\begin{equation*}l^C_{m\to j}(R_{\mathrm{eff}}, k) = \frac{m}{j}l_{j\to (j-m)}(R_{\mathrm{eff}}, k).\end{equation*}

The probability *p_i _*of observing a chain of size *M* (or greater) after *N_i_* introductions is one minus the probability that all clusters are smaller than *M*:

\begin{equation*}p_i = 1-\left(\sum^{M-1}_{j=1} l^C_{1\to j}\right) ^{N_i}.\end{equation*}

Given *N_i _*introductions per year, we can compute the expected number of years between events of size at least *M* as *1/p_i_.*

We use values of the dispersion parameter *k* derived from US national data in this estimation; although the estimate of *R_eff_* does not depend on *k*, the confidence interval does. Our central estimate of the dispersion parameter was 0.27, with 95% confidence interval of 0.18 to 0.41.^4^ Note that there is no evidence distinguishing California's MMR vaccination rate (90.7 ± 5.3) from the national vaccination rate (91.9 ± 0.9), so that US national estimates11 (with sensitivity analysis) provide a plausible choice of *k*.

To estimate an overall maximum likelihood effective reproduction number from an initial number of cases and a total cluster size, we use this assumed value of *k* and maximize the right hand side of the second equation above. To jointly estimate the maximum likelihood estimate of an effective reproduction number in the first generation and then in the subsequent rounds of transmission, we compute the probability that an initial number of cases gives rise to the next generation size (using the first equation), and that this number of cases in the next generation give rise to the remaining number of cases using the second equation. We assume independent transmissions, and add the log likelihoods for the first generation and the subsequent generations. Confidence intervals for these are computed using the likelihood ratio method.^14^ All computations were conducted using the statistics package R 3.1 for Ubuntu Linux (R Foundation for Statistical Computing, Vienna, Austria).

## Results

The outbreak began with individuals exposed in Disneyland between December 17 and 20, 2014; 40 Californians were believed exposed during that period.^3^ Subsequently, 91 additional cases (of the outbreak strain) are known to have occurred in the state. The epidemiology of the initial exposure has not been completely characterized, and even the number of index cases in December has not been reported. The initial exposure, occurring in a crowded theme park, may be considered a possible superspreading event.

When 40 first-generation measles cases produce 91 additional linked secondary cases (for a total chain size of 131 including a putative single index case), *R* is estimated to be 0.69 (95% CI: 0.48, 1.04; likelihood ratio CI assuming^7^ *k* = 0.27). If we use the 95% upper confidence bound for the estimated aggregation parameter *k*, 0.41, then we obtain a confidence interval for R of 0.50 to 0.98; using the 95% lower bound of *k*=0.18, we obtain a confidence interval of 0.45 to 1.12. Smaller values of *k* yield wider confidence limits still; *k*=0.05 provides a confidence interval of 0.34 to 1.71.

We conducted an additional sensitivity analysis in which we no longer assumed that the effective reproduction number was constant. Assuming that the approximately 48 cases reported in the two weeks immediately following the last of the 40 Disney-exposed cases to be the next generation of transmission outside Disneyland, we find maximum likelihood estimates of the effective reproduction number to be 1.20 (=48/40) (95% CI: 0.65, 2.45, assuming *k*=0.27) in the first generation and 0.47 (95% CI: 0.29, 0.79, assuming *k*=0.27) for the remaining transmission events. This difference is not statistically significant (*P*=0.09, likelihood ratio test). Assuming a smaller *k* value of 0.18 widens the confidence intervals on these estimates, and increases the *P*-value to 0.12.

Finally, if we assume 60 introductions per year into the US, and use the estimated effective reproduction number 0.52 and dispersion parameter 0.27 from the 2001-2011 data^4^, then we would expect clusters of size at least 147 (the size of the outbreak in the US, including the 131 California cases) to occur approximately once every 14200 years. A smaller *k* of 0.18 (the lower confidence limit from the earlier analysis) and larger *R_eff_* (0.60, the upper confidence bound) would yield a frequency of once every 140 years.

**Probability of observed transmission chain vs. reproduction number:**
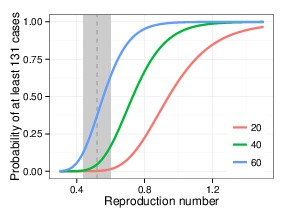
Probability of at least 131 cases (the chain size restricted to California) following 20, 40, or 60 first generation cases in California as a function of reproduction number *R*_eff_, with *k*=0.27. Gray vertical line and shading depict the estimated *R*_eff_ and 95% CI based on data for measles transmission in the United States from 2001–2011.

## Discussion

Except for the substantial initial transmission event that occurred within the Disney theme parks, the transmission of measles seen in the recent outbreak is relatively consistent with data from the past decade. In particular, amplification from 40 cases to 91 additional cases is consistent with subcritical transmission, with each case failing, on average, to replace itself, but playing out over several generations of transmission. Our primary estimate of the reproduction number, 0.69, is higher than the number obtained from US national data for 2001-2011 (0.52), but the difference is not statistically significant. Our second estimate (in which we allowed the effective reproduction number** to be larger in the first generation outside Disneyland) suggested the possibility of an effective reproduction number over one, but inaccuracy in classifying cases as being in this next generation, together with wide confidence bands, limits our ability to draw firm conclusions from this result.

Use of the Galton-Watson process with a negative binomial distribution accounts for heterogeneity in transmission, but we are limited in that our estimated dispersion parameter (*k*) was derived from an observation period (2001-2011) which may not be sufficient to characterize superspreading events. We found that the current outbreak does seem to be unusual based on our US national model of 2001-2011, in the sense that outbreaks as large or larger should occur with a frequency of less than once per century. Of course, such estimates are difficult to interpret in the absence of a prespecified analysis plan.

Our primary analysis also assumed a constant value for the reproduction number during the entire outbreak. However, the onset of rash of the first case was on Dec. 28, 2014, while the outbreak proper did not come to the attention of the public until Jan. 6^10^, so that at least for a few cases, more transmission may have been likely for several days than later in the outbreak, once widespread public awareness could contribute to rapid diagnosis. While we failed to find evidence of a change in the effective reproduction number, the results suggest that further investigation is warranted to determine whether or not there was more transmission during the earlier part of the epidemic. It should also be asked whether such transmission is due to clustering of unvaccinated individuals, or to a time lag in the intensification of contact investigation and public awareness. An alternative analysis suggested a more substantial decline.^15^

It is important to note that the epidemiology of recent years post-elimination illustrates that even with the absence of endemic measles, transmission can still occur with imported infections and susceptible individuals.^8^ High vaccination coverage remains the best method of measles control and prevention.^9^

## Competing Interests

All authors have confirmed that they have no financial, personal or professional competing interests to report.
